# Numerical Simulation of Thermal Fields and Microstructure Evolution in SLM of Fe_32_Cr_33_Ni_29_Al_3_Ti_3_ Alloy

**DOI:** 10.3390/mi16060694

**Published:** 2025-06-10

**Authors:** Xuyun Peng, Xiaojun Tan, Haibing Xiao, Wei Zhang, Liang Guo, Wei Tan, Jian Huang, Chaojun Ding, Yushan Yang, Jieshun Yang, Haitao Chen, Qingmao Zhang

**Affiliations:** 1 Sino-German Intelligent Manufacturing School, Shenzhen City Polytechnic, Shenzhen 518116, China; 19970975568@163.com (X.P.); 18620373551@163.com (W.T.); hejson@163.com (J.H.); 18617076210@163.com (C.D.); real210@163.com (Y.Y.); yangjieshun@126.com (J.Y.); a202415814644653@163.com (H.C.); 2 Intelligent Manufacturing and Equipment School, Shenzhen Institute of Information Technology, Shenzhen 518172, China; xiaohb@sziit.edu.cn (H.X.); wzhang@yeah.net (W.Z.); 3 Guangdong Provincial Key Laboratory of Nanophotonic Functional Materials and Devices, School of Information and Optoelectronic Science and Engineering, South China Normal University, Guangzhou 510006, China; guoliangchn@163.com (L.G.); zhangqm@scnu.edu.cn (Q.Z.)

**Keywords:** high-entropy alloy, selective laser melting, thermal simulation, microstructure evolution, FCC, BCC

## Abstract

Fabricating eutectic high-entropy alloys (EHEAs) via selective laser melting (SLM) presents significant potential for advanced structural applications. This study explores the microstructural evolution of Fe_32_Cr_33_Ni_29_Al_3_Ti_3_ EHEAs fabricated by SLM under varying laser powers. Electron backscatter diffraction (EBSD) analysis revealed that samples fabricated at 200 W exhibited approximately 70% face-centered-cubic (FCC) and 30% body-centered-cubic (BCC) phases. In comparison, those processed at 160 W showed an increased FCC fraction of 85% with a corresponding reduction in BCC content. Grain size measurements indicated that BCC grains were consistently finer than their FCC counterparts. Thermal simulations demonstrated that higher laser power produced deeper melt pools and broader temperature gradients. By correlating thermal history with phase diagram data, the spatial variation in BCC content was attributed to the differential residence time in the 1350–1100 °C range. This study represents one of the first attempts to quantitatively link local thermal histories with the evolution of dual-phase (FCC + BCC) microstructures in EHEAs during SLM. The findings contribute to the improved understanding and control of phase formation in complex alloy systems, providing valuable guidance for tailoring SLM parameters to optimize the phase composition and microstructure of EHEAs.

## 1. Introduction

High-entropy alloys (HEAs) have garnered increasing interest due to their remarkable mechanical properties, thermal stability, and corrosion resistance, making them promising candidates for applications in aerospace, energy, and other demanding industrial fields [1]. When combined with selective laser melting (SLM), an advanced additive manufacturing technique that enables the production of complex geometries with high precision and near-net-shape accuracy, the potential for fabricating high-performance HEA components is significantly expanded [2]. This integration of HEAs with SLM offers a powerful pathway for the development of next-generation structural materials.

Among various strengthening mechanisms employed in HEAs, such as particle strengthening [3], nanoprecipitation [4], and eutectic structure reinforcement [5], the eutectic approach has demonstrated particular promise. Eutectic high-entropy alloys (EHEAs) are characterized by phase compatibility and refined microstructures, offering an attractive combination of high strength and ductility, along with improved manufacturability [6,7]. Consequently, EHEAs fabricated via SLM have emerged as a focal point in recent HEA research. Notable examples include AlCoCrFeNi_2.1_ [8,9], CrFeCoNiAl_0.4_Ti_0.14_ [10], Al_0.7_CoCrFeNi_2.4_ [11], (FeCrNi)_94_Ti_3_Al_3_ [12], AlCoCrFeNi_2.5_ [13], and Al_18_Co_30_Cr_10_Fe_10_Ni_32_ [14], all of which have demonstrated impressive mechanical performance, with yield strengths exceeding 900 MPa and ultimate tensile strengths surpassing 1000 MPa.

Despite these advances, the mechanisms governing microstructure evolution during the SLM process, particularly in multi-phase and compositionally complex HEAs, remain insufficiently understood. Dual-phase systems such as FCC + BCC EHEAs are especially sensitive to thermal gradients and solidification conditions, making them more challenging to control. In this context, temperature field simulations have emerged as an effective approach to predict thermal behavior and correlate it with microstructure formation. Prior research has leveraged numerical modeling to study heat transfer and solidification phenomena in HEAs [15], stainless steels [16], and aluminum alloys [17], employing tools ranging from finite element analysis (FEA) to multi-physics and data-driven methods [18]. These efforts have provided valuable insights into melt pool dynamics, thermal gradients, solidification rates, and their influence on microstructural defects and mechanical properties.

However, few studies have simultaneously conducted phase-resolved EBSD analysis and location-specific thermal simulations to explain microstructure evolution in dual-phase HEAs. In particular, the relationship between localized thermal gradients and FCC/BCC phase formation remains poorly understood. The complex interplay of phase transformation, segregation, and microstructure development in such systems necessitates a deeper understanding to fully exploit the capabilities of SLM.

To address this gap, the present study investigates the microstructural evolution of a dual-phase EHEA with the nominal composition Fe_32_Cr_33_Ni_29_Al_3_Ti_3_, previously developed and characterized in our earlier work [19]. By combining temperature field simulations with experimental analysis under different laser power conditions, we aim to elucidate the thermal mechanisms that govern microstructure formation. The insights gained provide a foundation for optimizing SLM process parameters and designing next-generation HEA components with tailored properties.

The novelty of this study lies in its integrated approach to understanding thermal–microstructural evolution in a dual-phase EHEA (Fe_32_Cr_33_Ni_29_Al_3_Ti_3_) fabricated by SLM. Unlike prior studies that treat microstructure and simulation separately, we quantitatively correlate local thermal gradients and residence times with phase fraction and grain morphology using coupled EBSD analysis and thermal modeling. This not only provides mechanistic insight into phase transformation pathways but also enables more precise control of phase distribution in multi-phase HEAs. Furthermore, this alloy system is underexplored in SLM, and our results contribute foundational knowledge for tailoring EHEA properties through process design.

## 2. Materials and Methods

### 2.1. Sample Preparation

**Figure 1:**
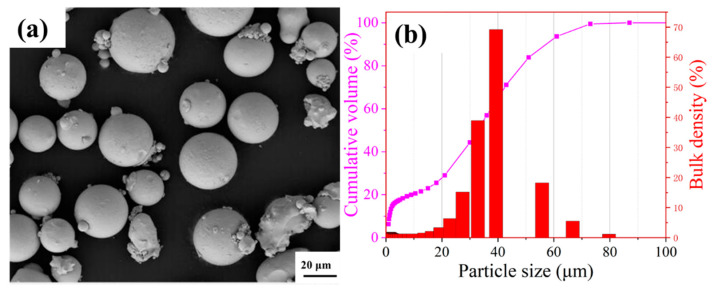
(**a**) SEM morphology; (**b**) particle size distribution of the Fe_32_Cr_33_Ni_29_Al_3_Ti_3_ high-entropy powder.

The pre-alloyed spherical Fe_32_Cr_33_Ni_29_Al_3_Ti_3_ high-entropy alloy (HEA) powder used for selective laser melting (SLM) was fabricated via vacuum induction melting followed by inert gas atomization, as shown in Figure 1a. The particle size distribution is characterized by D_10_ = 1.2 μm, D_50_ = 32.7 μm, and D_90_ = 57.2 μm, as illustrated in Figure 1b.

Cubic specimens of Fe_32_Cr_33_Ni_29_Al_3_Ti_3_ (10 × 10 × 10 mm^3^) were fabricated using a selective laser melting (SLM) system (HANS-100, Han’s Laser, Shenzhen, China), equipped with a 500 W fiber laser operating at a wavelength of 1070 nm and a focused beam diameter of 50 μm. All fabrication was performed in an argon atmosphere to prevent oxidation. A zigzag scanning strategy with a 67° interlayer rotation angle, 60 μm hatch spacing, and 30 μm layer thickness was employed.

**Figure 2:**
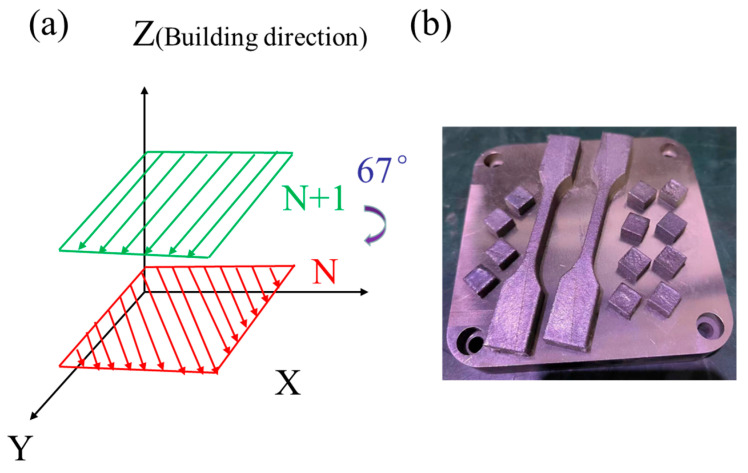
(**a**) Schematic illustration of the SLM building strategy and (**b**) SLM cubic and tensile testing specimens.

To examine the influence of laser power on microstructure, two processing conditions were used with identical scanning speeds of 1000 mm/s: one at 160 W (P160) and the other at 200 W (P200). The X–Y plane refers to the plane perpendicular to the build direction, while the X–Z plane lies parallel to the build direction, as illustrated in Figure 2a. All specimens (cubic and tensile testing specimens) were built on a 304 stainless steel substrate (Figure 2b).

### 2.2. Microstructural Characterization

Microstructural evaluation was conducted on the X–Z plane, which is particularly suited for assessing microstructure evolution, grain growth behavior, and heat-affected zone characteristics in the build direction [20]. Before analysis, standard metallographic preparation was carried out. Samples were ground with silicon carbide papers (400–1500 grit), followed by polishing with a 0.05 μm alumina suspension. Final polishing was performed for approximately 7 h using a 50 nm colloidal silica suspension to eliminate surface deformation.

After thorough rinsing with distilled water, electron backscatter diffraction (EBSD) measurements were performed using a field emission scanning electron microscope (Gemini 300, ZEISS, Oberkochen, Germany) equipped with an EBSD detector (Symmetry, Oxford Instruments, Oxford, UK). A step size of 0.18 μm was used during scanning, and data were processed using the Aztec Crystal 2.1.2 software suite.

Phase analysis was conducted using X-ray diffraction (XRD) on a D8 Advance diffractometer (Bruker, Karlsruhe, Germany) equipped with Cu Kα radiation. Scans were performed over a 2θ range of 20° to 120° at a rate of 10° per minute.

### 2.3. Tensile Testing

Uniaxial tensile tests were conducted at room temperature using a universal testing machine (Model EM6.304-L, 30 kN capacity, TSMT, Shenzhen, China) under a constant crosshead speed of 0.5 mm/min. The specimens, machined into dog-bone shapes with a gauge length of 25 mm, width of 5 mm, and thickness of 2 mm, were prepared following ASTM E1820 guidelines [21]. During testing, the loading direction was set perpendicular to the layer-wise build direction of the printed samples.

## 3. Numerical Simulations

To investigate the thermal behavior during the SLM process of Fe_32_Cr_33_Ni_29_Al_3_Ti_3_, transient temperature field simulations were conducted using Abaqus 2022 software. The simulation incorporated temperature-dependent material properties, a double-ellipsoidal heat source, phase change treatment, and appropriate boundary conditions to closely replicate experimental conditions.

### 3.1. Governing Equations

The transient heat conduction during laser processing is governed by the following nonlinear partial differential equation:

(1) $$\rho c\frac{\partial T}{\partial t}=\frac{\partial }{\partial x}\left(k\frac{\partial T}{\partial x}\right)+\frac{\partial }{\partial y}\left(k\frac{\partial T}{\partial y}\right)+\frac{\partial }{\partial z}\left(k\frac{\partial T}{\partial z}\right)+Q$$

where ρ is the material density (g/cm^3^), c is the specific heat capacity (J/(mole·K)), T is the temperature (K), *t* is the time (s), k is the thermal conductivity (W/(m·K)), and Q represents the internal heat source due to the laser.

**Figure 3:**
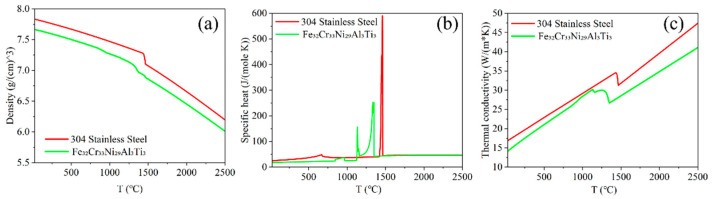
Temperature-dependent material properties of the 304 stainless steel substrate and Fe_32_Cr_33_Ni_29_Al_3_Ti_3_ alloy: density (**a**); specific heat (**b**); thermal conductivity (**c**).

The thermophysical properties of the HEA and 304 stainless steel substrate were obtained from JMATPro 15.0 simulations, as shown in Figure 3 [15]. Below the melting point, the powder was assumed to retain the specific heat of the bulk but with 60% of its density and only 1% of its thermal conductivity [22,23]. Above the melting point, liquid-phase properties were assumed to match those of the bulk alloy.

### 3.2. Laser Heat Source Model

**Figure 4:**
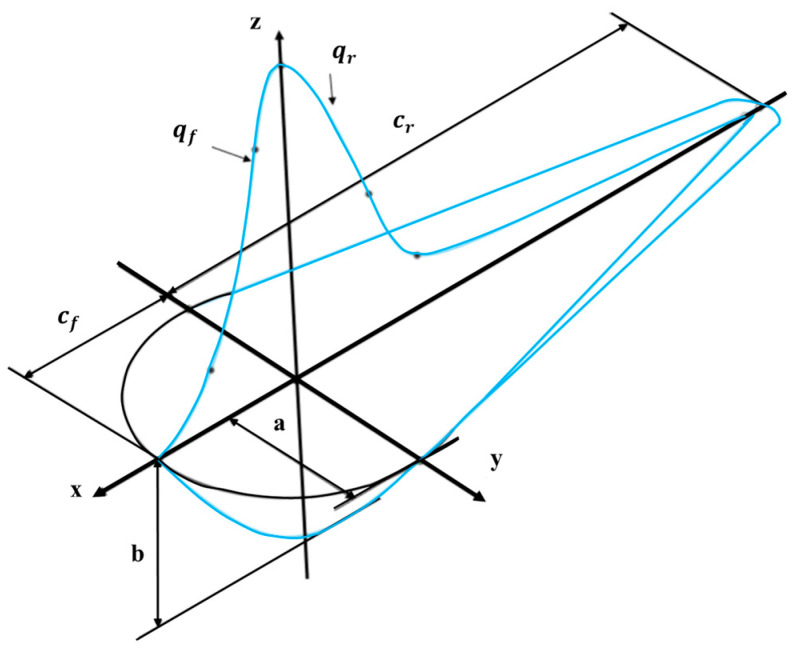
Schematic of the double ellipsoidal heat source model.

A double-ellipsoidal volumetric heat source was employed to model the laser energy input (Figure 4) [24]. The heat flux distribution within the ellipsoids is described as follows [25]:

(2) $$q_{4}\left(x,y,z\right)=\left\{\begin{array}{ll} \frac{6\sqrt{3}AP}{\pi \sqrt{\pi }ab_{r}c}exp\left.\left\{-3\left[\left(\frac{x^{2}}{a^{2}}\right)\right.+(\frac{y^{2}}{{b_{r}}^{2}})\right.+\left(\frac{z^{2}}{c^{2}}\right)\right], & x\geq 0\\ \frac{6\sqrt{3}AP}{\pi \sqrt{\pi }ab_{f}c}exp\left.\left\{-3\left[\left(\frac{x^{2}}{a^{2}}\right)\right.+(\frac{y^{2}}{{b_{f}}^{2}})\right.+\left(\frac{z^{2}}{c^{2}}\right)\right], & x<0 \end{array}\right.$$

where a, $b_{r}$, $b_{f}$, and c define the ellipsoid dimensions; A is the absorption coefficient (assumed to be 0.33 [26]); and P is the laser power (W).

### 3.3. Latent Heat Treatment

The phase change was handled using the enthalpy method. The total enthalpy H is defined as follows:

(3) $$H=\int_{T_{0}}^{T}c_{p}dT+f_{L}L$$

where $f_{L}$ is the liquid fraction, and L is the latent heat of fusion. The liquid fraction is determined by the following:

(4) $$f_{L}=\left\{\begin{array}{cc} 0, & T<T_{s}\\ \frac{T-T_{s}}{T_{L}-T_{S}}, & T_{s}<T<T_{L}\\ 1, & T>T_{L} \end{array}\right.$$

where $T_{s}$ and $T_{L}$ are the solidus and liquidus temperatures, respectively.

### 3.4. Boundary Conditions

Three boundary conditions were applied:

(1) Initial temperature:
  (5) $$T\left(x,y,z,t=0\right)=T_{0}$$
(2) Heat flux input:
  (6) q=−k∇T·n
(3) Surface losses (convection and radiation):

(7) $$q_{rad}=\sigma \varepsilon (T^{4}-T_{\infty }^{4})$$

(8) $$q_{conv}=h(T-T_{\infty })$$

where σ is the Stefan–Boltzmann constant, ε is the surface emissivity, h is the convective heat transfer coefficient, and $T_{\infty }$ is the ambient temperature.

### 3.5. Finite Element Model Setup

**Figure 5:**
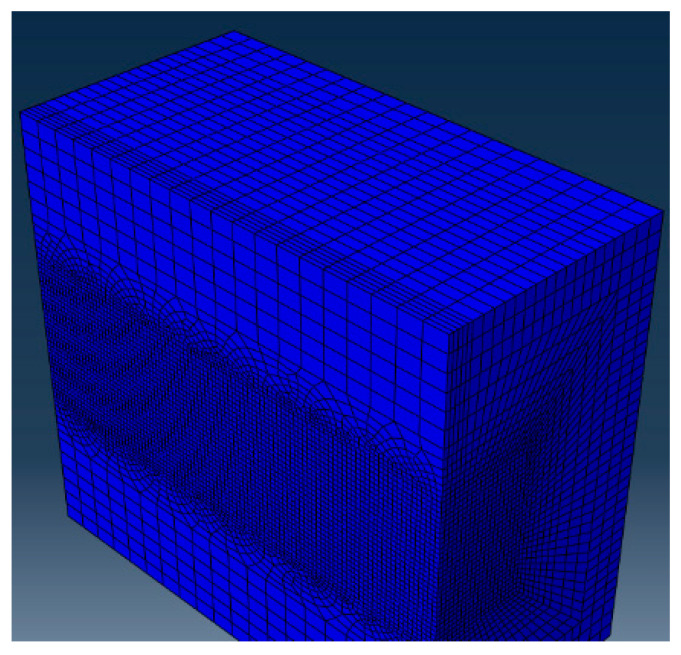
Meshing strategy of the finite element model showing fine and coarse mesh regions.

A 3D finite element model with dimensions 0.6 × 0.3 × 0.3 mm^3^ was constructed. The top 30 μm layer represented the powder layer. To ensure computational efficiency and accuracy, adaptive meshing was used. A fine mesh (6 × 6 × 6 μm^3^) was applied near the laser path, while coarser elements were used elsewhere (Figure 5).

## 4. Results and Discussion

### 4.1. XRD and SEM Analysis

**Figure 6:**
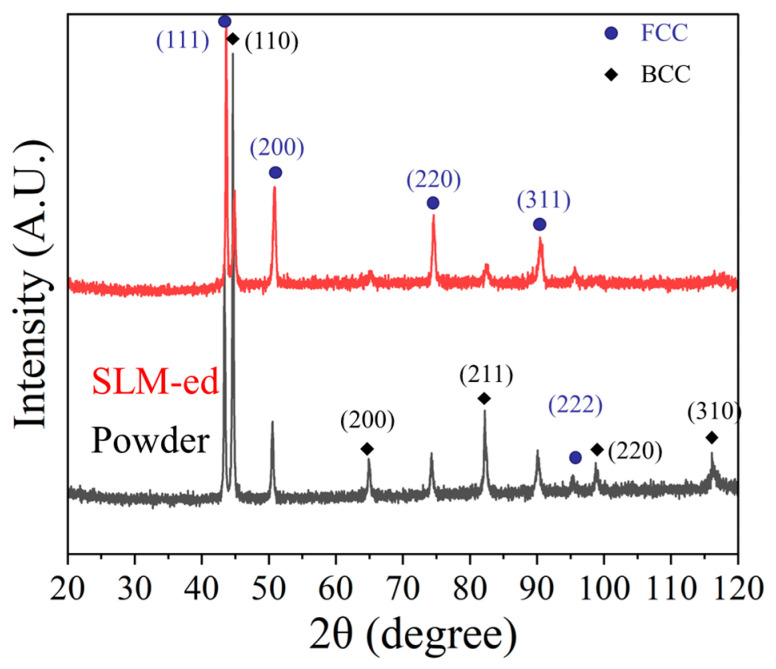
XRD patterns of HEA powder and sample P200.

The XRD analysis of the HEA powder and P200 is presented in Figure 6 to confirm the phase selection of the FCC and BCC dual phases.

**Figure 7:**
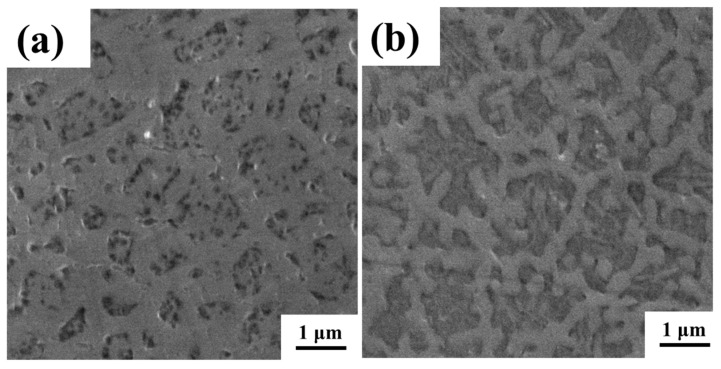
Microstructure of P160 (**a**) and P200 (**b**) observed by SEM.

The microstructure on the X-Y plane of P160 and P200 was observed by SEM, and the results are shown in Figure 7. Both show two colors: black and grey. The duplex structure of the alloy is illustrated.

### 4.2. EBSD Analysis of Phase Distribution and Grain Size

**Figure 8:**
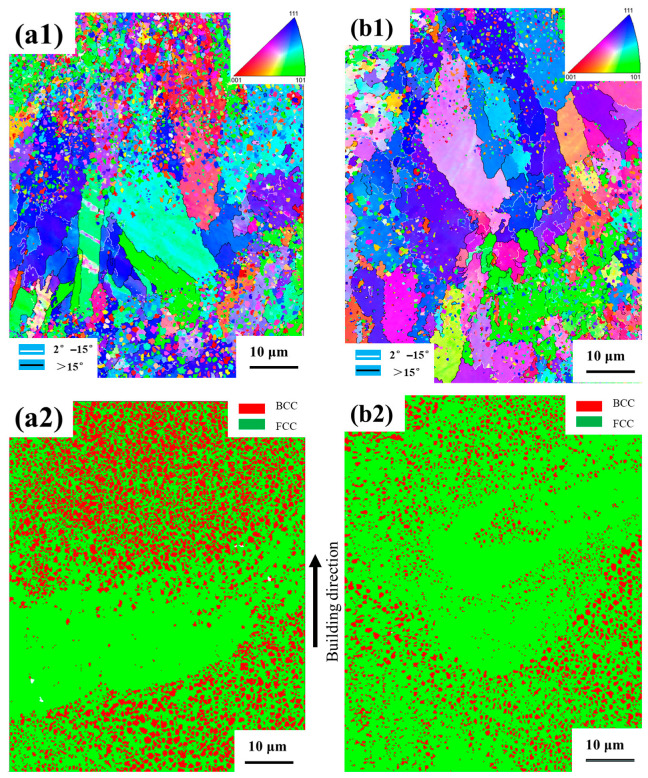
IPF map with grain boundary of P200 (**a1**) and P160 (**b1**); phase distribution of P200 (**a2**) and P160 (**b2**), along the building direction.

The EBSD phase maps of the X–Z plane for samples processed at 200 W (P200) and 160 W (P160) are shown in Figure 8. Figure 8(a1,a2) show the inverse pole figure (IPF) map with the grain boundary of the as-built HEA samples P200 and P160. The grains consist of equiaxed and columnar grains. At the center of the molten pool is a large number of equiaxed grains, accompanied by a significant number of columnar grains grown along the normal direction of the molten pool boundary. This phenomenon is more severe when closer to the bottom of the molten pool.

Both P200 and P160 exhibited dual-phase microstructures consisting of FCC and BCC phases. The spatial distribution of the BCC phase was highly nonuniform, being concentrated in the central and upper regions of the melt pool, while nearly absent near the bottom.

Quantitative phase analysis showed that the P200 sample contained approximately 70% FCC and 30% BCC phases, whereas the P160 sample consisted of 85% FCC and only 15% BCC. This suggests that higher laser power promotes BCC phase formation, likely due to increased thermal exposure and melt pool depth.

**Figure 9:**
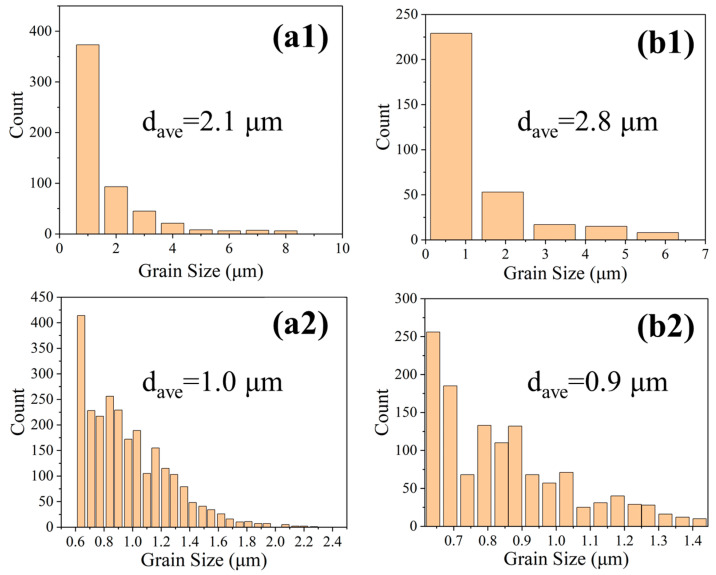
Distribution of the grain size of the FCC phase and the BCC phase of P200 (**a1**,**a2**) and P160 (**b1**,**b2**).

Grain size analysis revealed that BCC grains were consistently finer than FCC grains. In P200, the average FCC grain size was ~2.1 μm, while the BCC grains averaged ~1.0 μm, as shown in Figure 9(a1,a2). For P160, the FCC grain size increased to ~2.8 μm, and the BCC grains decreased slightly to ~0.9 μm, as shown in Figure 9(b1,b2). This indicates that lower energy input limits BCC formation and favors the growth of coarser FCC grains.

### 4.3. Spatial Temperature Field and Melt Pool Geometry

**Figure 10:**
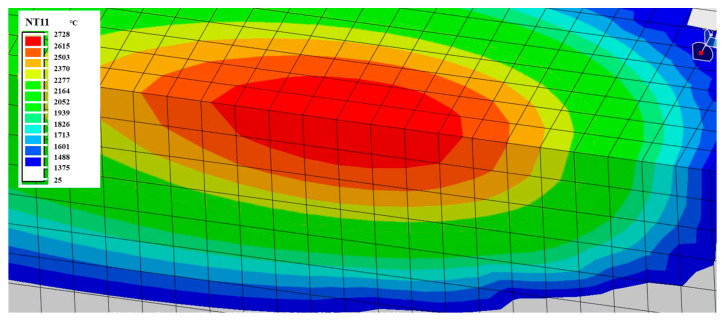
Simulated temperature field contour for P200 (200 W, 1000 mm/s).

The simulated transient temperature field for the P200 condition is shown in Figure 10. The temperature contours display an elliptical melt pool profile, which is symmetric about the scanning axis. This geometry reflects a typical SLM thermal distribution and aligns with previously reported experimental observations [27]. The shape and size of the melt pool directly influence cooling rates and subsequent phase evolution.

### 4.4. Comparative Thermal Profiles for P160 and P200

**Figure 11:**
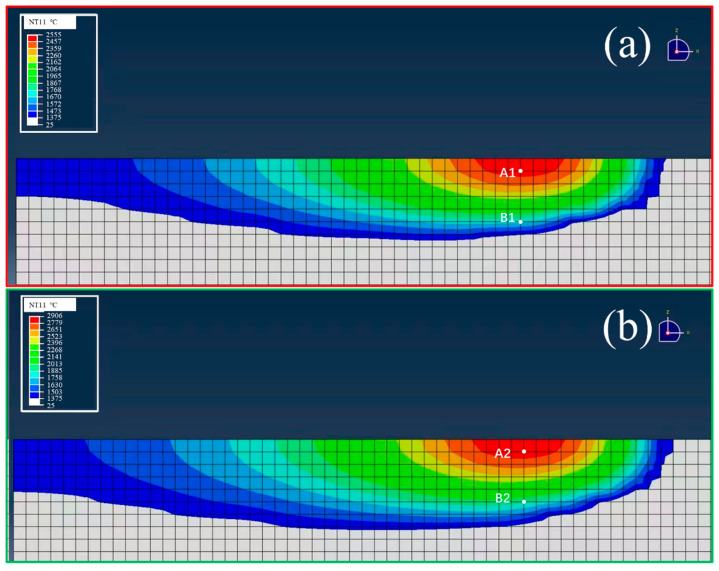
Temperature distribution in X-Z sections during SLM: (**a**) P160, (**b**) P200 (A1, A2 are points at the center, and B1, B2 are points at the bottom of the molten pools).

Figure 11 compares the X–Z temperature profiles of the two laser power conditions. The higher power (P200) produced a significantly deeper and wider melt pool, resulting in a broader thermal gradient. In contrast, the P160 profile is more localized, with a shallower melt pool and steeper thermal gradient.

These differences are critical, as thermal gradients affect solidification rates, undercooling, and the competitive growth of FCC vs. BCC phases. Larger melt pools with prolonged residence times favor increased BCC content and grain growth [28].

The depth of the simulated melt pool of P160 is about 37 μm in Figure 11a, which is very close to the depth measured by EBSD in Figure 8(b1), which is about 40 μm. Also, the depth of the simulated melt pool of P200 is about 45 μm in Figure 11b, which is very close to the depth measured by EBSD in Figure 8(a1), which is about 50 μm. These verified the degree of agreement between the simulation results and the experimental data.

### 4.5. Microstructure Evolution and Thermodynamic Interpretation

**Figure 12:**
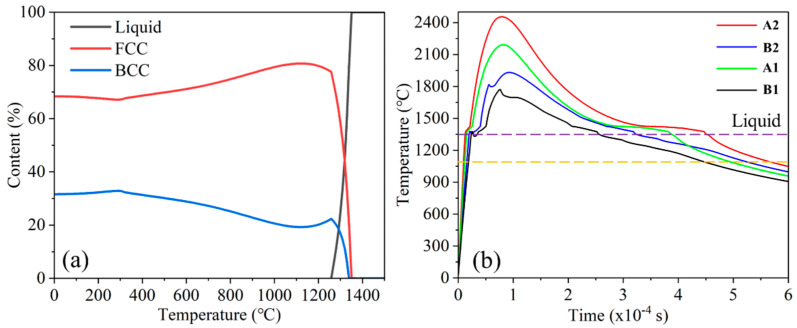
(**a**) Temperature curves of A1, A2, B1, B2; (**b**) phase diagram of Fe_32_Cr_33_Ni_29_Al_3_Ti_3_ EHEA (the dashed yellow line represents 1100 °C).

The equilibrium phase diagram for Fe_32_Cr_33_Ni_29_Al_3_Ti_3_ (Figure 12a) shows that solidification begins around 1350 °C with preferential FCC formation. BCC nucleation becomes significant below ~1250 °C. The FCC phase fraction peaks near 1100 °C, consistent with the observed microstructures.

To understand phase formation dynamics, temperature–time curves were extracted at four locations: A1, A2 (center), and B1, B2 (bottom) (Figure 11). Table 1 summarizes the residence times in critical temperature intervals (1350–1250 °C and 1250–1100 °C).

The bottom locations (B1, B2) experienced shorter times in the 1350–1250 °C range, limiting BCC nucleation. Conversely, the central points (A1, A2) had longer residence times, promoting BCC phase development. However, in the lower energy P160 condition, sharper cooling gradients resulted in a higher BCC phase fraction but a smaller grain size. For P200, prolonged exposure allowed BCC grains to grow larger despite fewer nucleation sites.

These results demonstrate how subtle variations in thermal history can modulate the phase content and grain morphology in SLM-processed EHEAs.

### 4.6. Mechanical Properties and Strengthening Mechanism

**Figure 13:**
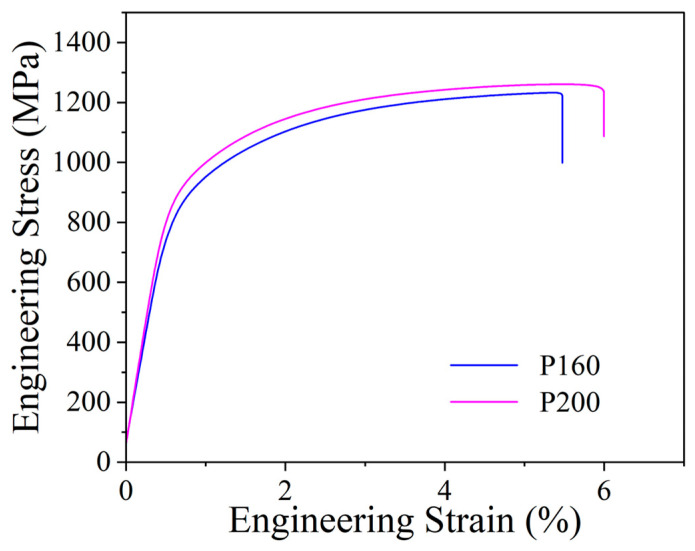
Tensile stress–strain curves at room temperature of P160 and P200.

The room-temperature tensile stress–strain curves for P160 and P200 are shown in Figure 13. The P160 sample exhibited a yield strength of 881 MPa, an ultimate tensile strength (UTS) of 1233 MPa, and an elongation of 4.7%. The P200 sample showed slightly higher values: 910 MPa yield strength, 1273 MPa UTS, and 6.0% elongation.

These mechanical differences can be attributed to microstructural characteristics (the BCC and FCC phase composition, grain size, and distribution). Phase fraction: Higher BCC content contributes to strength via its intrinsic hardness and impedes dislocation motion. The P200 sample, with higher BCC content, benefits from enhanced strength due to the inherent brittleness and high resistance to dislocation motion of the BCC phase [29]. Grain size: Finer BCC grains provide Hall–Petch strengthening, while coarser FCC grains in the lower power sample limit strengthening [30]. The larger BCC grain size in P200 may partially reduce the strength. Phase distribution and connectivity: The discontinuous BCC domain in the P160 sample reduces load transfer efficiency, whereas more continuous BCC domains in P200 enhance strengthening at the cost of some ductility [31].

In summary, the mechanical performance is governed by a complex balance between phase fraction, grain size, and phase distribution, all modulated by laser processing parameters.

## 5. Conclusions

In this study, the microstructural evolution and thermal behavior of a Fe_32_Cr_33_Ni_29_Al_3_Ti_3_ eutectic high-entropy alloy (EHEA) fabricated by selective laser melting (SLM) were systematically investigated through a combination of experimental characterization and numerical simulation. The key conclusions are as follows:

(1) Microstructural Variation: EBSD analysis revealed that increasing the laser power from 160 W to 200 W resulted in a higher fraction of the BCC phase (from 15% to 30%) and smaller FCC grain sizes. The BCC phase was primarily located in the upper melt pool regions, while nearly absent at the bottom.

(2) Thermal Field Simulation: A 3D transient temperature field model incorporating a double-ellipsoid heat source and temperature-dependent properties accurately captured the thermal profiles during SLM. The elliptical melt pool shape and thermal gradients predicted by the model were consistent with experimental observations.

(3) Melt Pool and Phase Formation: Residence time analysis showed that the bottom regions of the melt pool experienced shorter durations in the critical 1350–1250 °C range, limiting BCC nucleation. In contrast, longer residence times in central regions facilitated BCC formation. These findings directly correlated with the observed spatial distribution of the BCC phase.

(4) Process–Structure Relationship: The volumetric energy density (VED) influenced both the phase composition and grain size. Higher laser power (P200) led to larger BCC grains and improved ductility, while lower power (P160) favored finer microstructures and increased the BCC fraction due to rapid cooling.

This work demonstrates how integrated thermal simulation and microstructural analysis can guide the optimization of SLM parameters to tailor phase distribution and mechanical properties in EHEAs. These insights contribute to the broader understanding of process–structure–property relationships in the additive manufacturing of multi-principal-element alloys.

Overall, this work offers a novel framework for linking process parameters, simulated thermal fields, and experimentally observed microstructure in EHEAs, paving the way for more predictive SLM process design in multi-phase alloy systems.

## Figures and Tables

**Table 1:** Residence time of A1, A2, B1, B2 in each temperature zone.

Location	Residence Time (1350–1250 °C) (×10^−4^ s)	Residence Time (1250–1100 °C) (×10^−4^ s)
A1	0.88018	0.13402
A2	0.80276	0.28524
B1	0.3487	1.50978
B2	0.27612	1.66762

## Data Availability

The data that support the findings of this study are available from the corresponding author upon reasonable request.
